# Gut fungi are associated with human genetic variation and disease risk

**DOI:** 10.1371/journal.pbio.3003339

**Published:** 2025-09-02

**Authors:** Emily P. Van Syoc, Emily R. Davenport, Seth R. Bordenstein

**Affiliations:** 1 One Health Microbiome Center, Huck Institutes of the Life Sciences, The Pennsylvania State University, University Park, Pennsylvania, United States of America; 2 Department of Biology, The Pennsylvania State University, University Park, Pennsylvania, United States of America; 3 Department of Entomology, The Pennsylvania State University, University Park, Pennsylvania, United States of America; Johns Hopkins University Bloomberg School of Public Health, UNITED STATES OF AMERICA

## Abstract

Human genetic determinants of the gut mycobiome remain uninvestigated despite decades of research highlighting tripartite relationships between gut bacteria, genetic background, and disease. Here, we present the first genome-wide association study on the number and types of human genetic loci influencing gut fungi relative abundance. We detect 148 fungi-associated variants (FAVs) across 7 chromosomes that statistically associate with 9 fungal taxa. Of these FAVs, several occur in the protein-coding genes *PTPRC*, *ANAPC10*, *NAV2*, and *CDH13*. Additional FAVs link to tissue-specific gene expression as fungi-associated expression quantitative trait loci. Notably, the relative abundance of gut yeast *Kazachstania* associates with genetic variation in *CDH13* encoding T-cadherin, a protein linked to cardiovascular disease. *Kazachstania* forms a causal relationship with cardiovascular disease risk in a mendelian two-sample randomization analysis. These findings establish previously unrecognized connections between human genetics, gut fungi, and chronic disease, broadening the paradigm of human-microbe interactions in the gut to the mycobiome.

## Abstract 2

In contrast to decades of research on gut bacteria, human genetic determinants of the gut fungal community (mycobiome) remain understudied. This investigation presents the first GWAS on the number and types of human genetic loci that influence the relative abundance of gut fungi and links them to disease risk.

## Introduction

Human genetic variation impacts the abundance and diversity of gut microorganisms [1–3] that can triangulate with risk and development of chronic diseases [3,4]. The vast majority of human genome-wide and phenome-wide association studies on the microbiome (mbGWAS and mbPheWAS, respectively) focus solely on bacterial members [1,3,5,6]. For example, the most consistent and recurring gene-microbe associations are between the lactose digestion *LCT/MCM6* genomic region and the gut genus *Bifidobacterium* in wide-ranging cohorts [6,7]. Nonbacterial fractions of the gut microbiome remain understudied, especially gut fungi that constitute the “gut mycobiome”. Widespread perception of gut fungi as diet-derived transient passengers through the gastrointestinal tract [8,9] has hindered the investigation of how fungi assemble into a complex, multidimensional community, even with mounting evidence that gut fungi underpin human diseases [10,11] and gut inflammation [12,13]. Thus, establishing influences of human genetic variation on the gut mycobiome in a fungal-mbGWAS can evaluate the range of interactions beyond pathology. Here we interrogate the number and types of human genetic loci that influence gut fungal composition and chronic disease risk.

We leverage data from paired human genotypes and mycobiome profiles to identify fungi-associated variants (FAVs) whereby human genomic variation associates with variation in fungal communities. We then test whether these linkages between genetic loci and gut fungi in turn affect human disease risk. Determining whether human genetics simultaneously associates with differential microbial abundance and disease risk is a central challenge to resolve with substantive potential for personalized diagnostics and/or biotherapeutics. Taken together, this work advances the canonical, two-dimensional focus on human genetics and gut bacteria to the gut fungal biosphere.

## Results

### Human FAVs associate with gut fungal relative abundance

**Fig 1:**
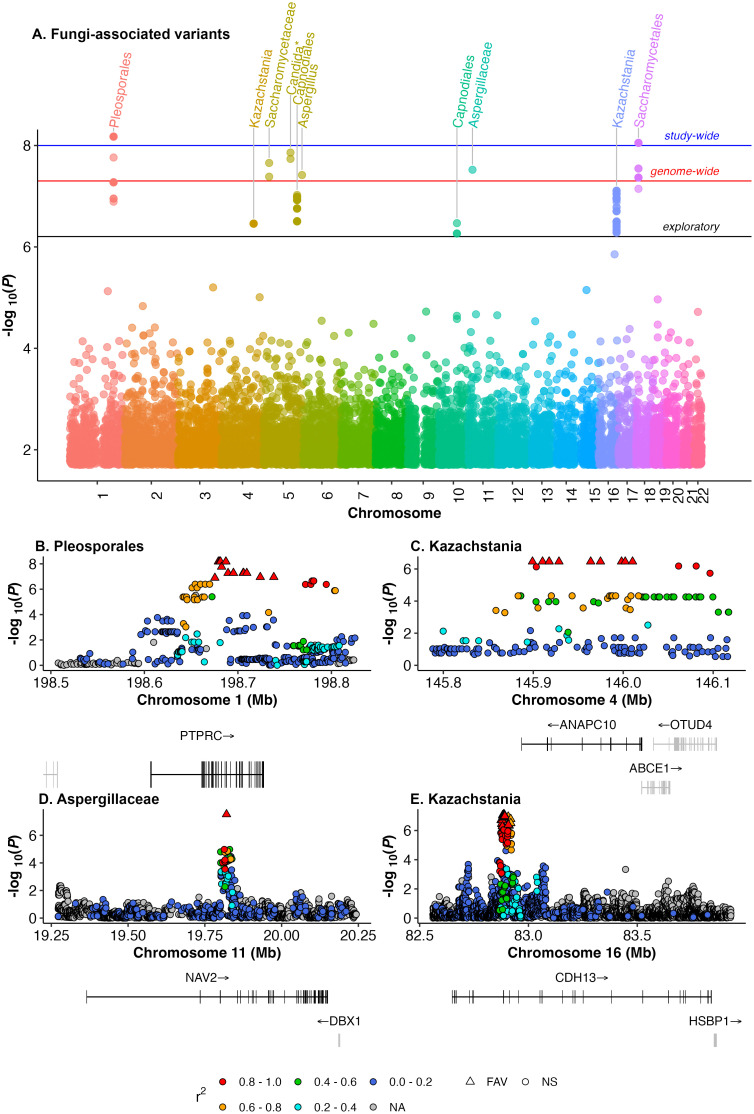
Human fungi-associated variants (FAVs) associate with 9 fungal taxa and overlap protein-coding genes. **(A)** Manhattan plot shows all FAVs and their associated fungal taxa at the three significance levels (exploratory, black; genome-wide, red; study-wide, blue). **(B–E)** FAVs (triangles) locate in four protein-coding genes, shown in black with exons in vertical lines. Points are colored by linkage disequilibrium score (*r*^2^). The protein-coding gene linked to each FAV is colored in black with exons in vertical lines. Abbreviations: FAV, fungi-associated variant; NS, not significant. The data underlying this figure can be found at https://doi.org/10.5281/zenodo.15659049.

We use paired gut mycobiome [14] and human genome [15] data from the Human Microbiome Project [16] (HMP, *n* = 125) to characterize genome-wide FAVs following methods established for gut bacterial GWAS [7,15,17]. We model each of ~5M human genetic variants that passed quality filtering for relationships with 44 prevalent fungal taxa that are present in at least 30% of individuals (S1 Fig). 148 FAVs (140 SNPs and 8 structural variants) across 7 chromosomes statistically associate at varying significance thresholds with 9 fungal taxa (Fig 1A). These fungal taxa include the genera *Aspergillus*, *Candida*, and *Kazachstania*; the families Saccharomycetaceae, a candidate Saccharomycetales family, and Aspergillaceae; and the orders Pleosporales, Capnodiales, and Saccharomycetales.

Specifically, 10 variants (9 SNPs and 1 structural variant on 2 chromosomes) associate with 2 fungi that meet a study-wide False Discovery Rate (FDR) correction across all variants and fungal taxa of *Q* < 0.2. Twenty-four variants (23 SNPs and 1 structural variant across 4 chromosomes) associate with 7 fungal taxa that meet genome-wide significance (*P* < 5 × 10^−8^). An additional 124 variants (117 SNPs and 7 structural variants across 6 chromosomes) associate with 4 fungal taxa that demonstrate significant associations at an exploratory significance threshold [1] of *Q* < 0.05 after FDR correction within each fungal taxa (S1 Table). Many of the 148 FAVs are in linkage disequilibrium with other FAVs on the same chromosome (S2 Fig).

Nearly half of all FAVs (*n* = 68) overlap the genomic coordinates of four protein-coding genes; *PTPRC* (ENSG00000081237; Fig 1B), *ANAPC10* (ENSG00000164162; Fig 1C), *NAV2* (ENSG00000166833; Fig 1D), and *CDH13* (ENSG00000140945; Fig 1E and S2 Table) whose functions have relevance to adaptive and innate immunity, kidney and liver neurology, and cardiovascular health. Similar to human bacterial microbiome-associated variants [3], these FAVs tend to be intronic or noncoding with a few annotated as 3′ downstream, 5′ upstream, or in the 3′ UTR region (S3 Fig). These 68 FAVs in noncoding regions of protein-coding genes in turn associate with three fungal taxa described further below.

First, the order Pleosporales associates with FAVs in the *PTPRC* gene, a transmembrane glycoprotein, specifically a tyrosine phosphatase receptor type C (UniProt P08575) termed CD45 (Fig 1B). It plays a central role in T-cell activation and impacts autoimmune conditions, cancers, and fungal infections [18]. CD45 localizes on the surface of T- and B-lymphocytes and interacts with Dectin-1, a pattern recognition receptor that responds to fungal beta-glucans [18]. Second, the fungal family Aspergillaceae associates with FAVs in the *NAV2* gene (UniProt Q8IVL1), a neuron navigator that guides axon growth during neural development [19] (Fig 1D). *NAV2* is broadly expressed across tissues, including the small and large intestines and exhibits pleiotropic functions, but is implicated in colorectal cancer and rheumatoid arthritis, suggesting an immunomodulatory connection [20,21]. Moreover, in *Caenorhabditis elegans*, *NAV2* expression shifts under varying exposures to bacterial pathogens, supporting a role in microbial response [22]. Third, the genus *Kazachstania* associates with FAVs located in two distinct genes, *ANAPC10* (Fig 1C) and *CDH13* (Fig 1E). *ANAPC10* (UniProt Q9UM13) codes for a core subunit of the anaphase-promoting complex subunit 10, which is a component of an E3 ubiquitin ligase that promotes anaphase in the cell cycle and may mediate innate immunity via NLRP3 inflammasome activation in a cell cycle-dependent manner [23,24]. Notably, *CDH13* codes for T-cadherin (UniProt P55290; also known as cadherin 13), which is a cell adhesion protein with diverse roles including binding adiponectin. Adiponectin is a circulating chemokine that regulates cholesterol circulation. *CDH13* is expressed in multiple tissue types, including cardiomyocytes, blood vessels, and intestines [25], and disruption of T-cadherin’s interactions with adiponectin increases the risk of cardiovascular disease [25–27].

### FAVs triangulate fungal abundance with variation in gene expression

**Fig 2:**
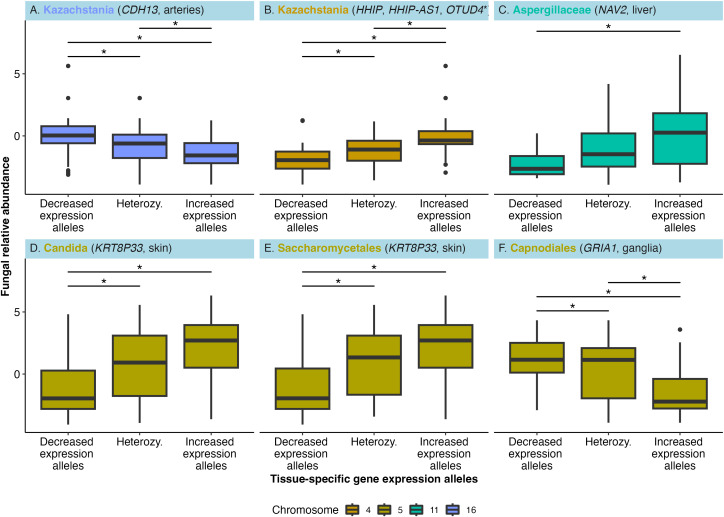
Fungal relative abundance links to FAV-eQTLs and variation in tissue-specific relative gene expression. **(A–F)** Individual genotypes for each FAV-eQTL associate with increased relative gene expression, decreased relative gene expression, or are heterozygous and possess one increased and decreased expression allele. Center-log transformed fungal relative abundance statistically associates with FAV-eQTLs in a genome-wide association study that in turn links to variation in relative gene expression for the gene shown in parentheses. Asterisks denote a statistically significant difference in fungal relative abundance between genotypes for each fungal taxa (Kruskal–Wallis with Dunn’s post hoc test, *P* < 0.05). Colors correspond to the chromosome of each FAV-eQTL. (B*) *Kazachstania* abundance associates with the expression of *HHIP* in nerves, *HHIP-AS1* in skin, and *OTUD4* in fibroblasts. Some data for this figure is restricted due to the use of protected human genomics data. Available data underlying this figure are at: https://doi.org/10.5281/zenodo.15659049.

We next investigate the potential for FAVs to influence relative gene expression via the colocalization of FAVs with expression quantitative trait loci, defined as FAV-eQTLs. Using the Genotype-Tissue Expression (GTEx) database spanning 50 tissue types [28], 82 out of 144 FAVs annotated in GTEx are FAV-eQTLs that associate with tissue-specific gene expression in 8 genes (S3 Table) and 4 fungal taxa. First, the relative abundance of the genus *Kazachstania* inversely correlates to FAV-eQTL-associated relative expression of *CDH13* in arteries (*n* = 25 FAV-eQTLs, Fig 2A), i.e., the allele associated with increased relative abundance of *Kazachstania* associates with decreased relative gene expression in the host. *Kazachstania* relative abundance also positively associates with 3 FAV-eQTL-associated genes (*n* = 10 FAV-eQTLs for all three genes) that are upregulated in fibroblasts (*OTUD4*), nerves (*HHIP*), and skin (*HHIP-AS1*) (Fig 2B). The remaining FAV-eQTLs associate with four additional fungal taxa and the expression of three genes (Aspergillaceae family and *NAV2* relative expression in the liver, Fig 2C; *Candida* genus and a candidate Saccharomycetales family and *KRT8P33* expression in skin, Fig 2D and 2E, and the Capnodiales order and *GRIA1* relative expression in basal ganglia, Fig 2F). Notably, the gut fungi that link to FAV-eQTLs are lineages that include human pathogens, including *Kazachstania*, *Aspergillus*, *Candida*, and the Capnodiales order that includes pathogens such as *Cladosporium* [29]*.* Taken together, these findings support interactions between gut fungi and human genes that may regulate cardiovascular health and antifungal immunity.

### *Kazachstania* links to cardiovascular disease risk variants

To triangulate FAV-fungi-disease relationships, we analyze phenome-wide associations of all 148 FAVs with 1,149 phenotypes from more than 400,000 individuals in the UK Biobank TOPMed-imputed PheWeb dataset [30]. Two *Kazachstania* FAVs, rs12929586 and rs12149890 (both located in intronic regions of *CDH13* in linkage disequilibrium with each other), associate with ischemic cardiovascular disease at Bonferroni-corrected phenome-wide significance (PheWEB effect size 0.039 ± 0.0095, *P* = 3.5 × 10^−5^). Ischemic cardiovascular disease (i.e., coronary heart disease or coronary atherosclerosis) occurs because there is loss of blood flow to a region of the heart caused by narrowed or blocked coronary arteries [26]. These FAVs additionally nominally associate with coronary atherosclerosis (0.042 ± 0.012, *P* = 3.4 × 10^4^) and unspecified cardiovascular disease (0.044 ± 0.013, *P* = 7.3 × 10^−4^).

**Fig 3:**
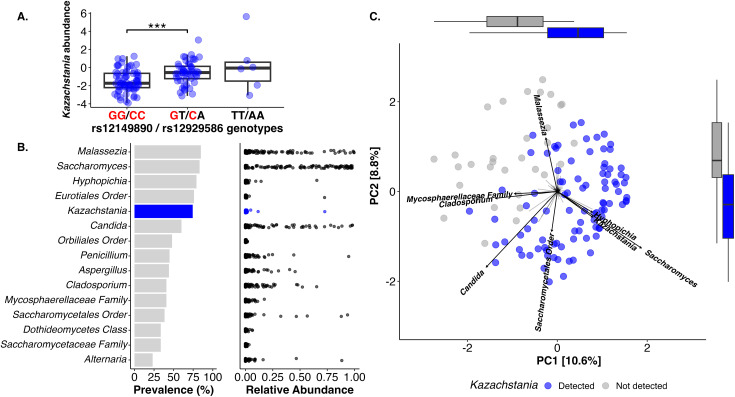
The gut yeast *Kazachstania* associates with heart disease risk alleles and altered gut mycobiome composition in healthy adults. **(A)** *Kazachstania* abundance in center-log coordinates is lower in individuals who harbor the risk alleles for ischemic cardiovascular disease, colored in red. **(B)** The prevalence (left) and relative abundance (right) varies among the 15 most prevalent fungal genera in the Human Microbiome Project cohort. **(C)** Principal component analysis distinguishes the gut mycobiome composition in individuals who harbor *Kazachstania* (blue) compared to individuals without detectable *Kazachstania* (gray). The top 8 fungal genera that contribute to PCA taxa loadings are shown in black italicized text with arrow lengths that approximately correspond to the contribution of distinguishing principal component axes. Boxplots on the top and right show the distribution of PC1 and PC2 coordinates, respectively, based on the presence of *Kazachstania*. Some data for this figure is restricted due to the use of protected human genomics data. Available data underlying this figure are at: https://doi.org/10.5281/zenodo.15659049.

Decreased *Kazachstania* abundance notably occurs in individuals homozygous for the cardiovascular disease risk alleles (*P* = 0.002; Fig 3A). We validated the relationships between rs12149890, rs12929586, and heart disease in a second GWAS using both the UK Biobank and the CARDIoGRAMplusC4D cohort comprised of 547,261 individuals [31] (beta = 0.0334, *P* = 6.1 × 10^−6^). Two-sample Mendelian randomization analysis further demonstrates a causal link between *Kazachstania* and cardiovascular disease using an outcome GWAS on coronary artery disease (Wald ratio *b* = −0.02, Bonferroni-adjusted *P* = 0.011). Moreover, the findings are consistent with a prior study where *Kazachstania* negatively correlates to cardiovascular disease risk scores and carotid artery thickness, a diagnostic measure of atherosclerosis [32].

Together, the findings support a previously unidentified, triadic relationship between *Kazachstania*, FAVS rs12149890 and rs12929586, and susceptibility to cardiovascular disease. The genetic variations occur in *CDH13*, which is implicated in cardiovascular disease by multiple GWAS [26,33]. As aforementioned, T-cadherin is the protein product of *CDH13* and regulates circulating adiponectin, an antiatherogenic chemokine secreted by adipose tissue that regulates high-density lipoprotein formation. Thus, variants in the *CDH13* gene are pro-atherogenic that can, in turn, heighten cardiovascular disease risk [26,33]. Furthermore, *CDH13* variants associated with gut bacteria in two independent microbiome GWAS [34,35], providing a candidate mechanism for gut bacteria–fungi interactions that may interface with the heart–gut axis [36]. Interactions between gut bacterial and fungal taxa and/or metabolites are an underexplored topic by which gut inhabitants may contribute to human disease.

*Kazachstania* is an enigmatic and understudied yeast that is emerging as a key gut constituent of medical importance [37,38]. In the HMP cohort, six fungal Operational Taxonomic Units (OTUs) comprise the *Kazachstania* genus, including *K. barnettii*, *K. exigua*, *K. servazzii*, and *K. psuedohumilis*, in addition to two unnamed species. At the genus level, it is highly abundant, ranking 14th of 175 among fungal genera in count abundance after rarefaction (mean relative abundance 1.1 ± 6.6%). It is also prevalent and is detected in 74% of the individuals (Fig 3B).

Notably, we report that the presence of *Kazachstania* in this population associates with a distinct mycobiome composition compared to individuals without detectable *Kazachstania* (PERMANOVA of Bray–Curtis, genus-level distances *R*^2^ = 0.17, *P* < 0.001; Fig 3C). We validated this association by reanalyzing an independent cohort of healthy Chinese adults where *Kazachstania* is highly prevalent [39] (S4 Fig and S4 Table). In datasets with low *Kazachstania* prevalence (present in fewer than 30% of individuals), the pattern was not evident due to low statistical power. Among demographic and clinical variables including age, gender, self-reported race, systolic blood pressure, body mass index, tobacco use, and history of either cardiac (*n* = 6) or gastrointestinal disease (*n* = 24), *Kazachstania* relative abundance was positively associated with only participant age (*F*_1,116_ = 4.02, *P* = 0.047; S5 Table). Relative abundance variation of *Kazachstania* across age may interplay with the presence/absence of cardiovascular disease, which is more common in elderly populations [40].

## Discussion

Despite a growing body of evidence from genome-wide (mbGWAS) and phenome-wide association studies (mbPheWAS) that reveal human genetic variation links with the composition of gut bacteria and disease risk [1–3,7], there is a striking knowledge gap on human gut fungi. Nascent research indicates that the gut mycobiome modulates enteric and systemic inflammation [12], and perturbed mycobiome states associate with human disease analogously to bacterial “dysbiosis” [10]. Here, we leverage the only datasets that link gut fungi with human genotyping to investigate mycobiome-by-genome relationships.

This first-in-kind fungal mbGWAS detects 148 fungi-associated variants (FAVs) linked with nine fungal taxa. Post hoc analyses identify FAVs that both overlap with protein-coding genes and associate with tissue-specific gene expression (FAV-eQTLs), suggesting triadic interactions with human genetic background, gut fungi, and antifungal immunity. Notably, all gut fungi that statistically link to FAV-eQTLs are lineages with known human pathogens. FAVs that associate with the gut yeast *Kazachstania* consistently link with genetic variation in *CDH13* which codes for T-cadherin and phenotypes of cardiovascular disease risk. Mendelian two-sample randomization and prior clinical evidence [32] further implicate *Kazachstania* and genetic variation in *CDH13* to cardiovascular disease.

First described as a *Saccharomyces* and later *Candida*, *Kazachstania* was recently assigned its own genus, but species complex names in the literature can be interchanged [37,38,41]. While emerging reports caution that *Kazachstania* species is an emerging pathogen that may coinfect with *Candida* [37,38,41]*,* studies also suggest that *Kazachstania* protects against invasive candidiasis [42]. More recently, *Kazachstania* was found to be a key gut commensal in mice where it regulates innate immunity [13]. These findings contextualize *Kazachstania* as a newly described gut inhabitant that may play important roles in the human gut mycobiome with consequences for health and disease.

There is a global paucity of gut mycobiome characterizations that will necessitate resources to expand our understanding. We used the only paired genotyping and mycobiome sequencing data from the HMP cohort to conduct this first-in-kind fungal mbGWAS. Similarly to the early efforts in identifying gut bacteria-associated human genetic variants [7,17], our findings from a limited sample size form the first list of nominal FAVs for future benchmarking across global human cohorts. On the host side, larger sample sizes are necessary to identify FAVs with lower minor allele frequencies and smaller effect sizes; and studies across diverse populations are needed to reveal population-specific associations. On the fungal side, constraints of short-read amplicon sequencing currently preclude strain- or pangenome-resolution analyses, which could amplify these findings beyond aggregated taxonomy (e.g., genus level) as we present here. Expanding this framework across larger, more diverse human cohorts and higher fungal resolution will be an exciting next step to advance the fungal biosphere into precision medicine. Together, these findings support a multikingdom view of gut microbes linked with human genetic variation and disease risk. As such, human genetic influences on gut fungi may offer opportunities and challenges for precision diagnostics, especially as increasingly large and geographically diverse datasets of human microbiomes come to fruition.

## Methods

### Human research participants

All data used in this study are previously published and available to researchers from the Sequence Read Archive or dbGaP (see Data availability statement). Protocols for the HMP were approved by institutional review boards at each clinical site and are available in the original publications [14–16,43]. The HMP cohort is the only resource among a paucity of mycobiome studies with marker sequencing for gut fungi (Internal Transcribed Spacer region, a ribosomal marker gene for eukaryotes that taxonomically resolves fungi to the genus level similarly to 16S for bacteria; first published by Nash and colleagues [14]) paired with host genotyping (first published by Kolde and colleagues [15]). The Pennsylvania State University Institutional Review Board exempted the use of this data from institutional review (STUDY00023406).

### Fungal amplicon sequencing data

An amplicon processing bioinformatics pipeline was applied to the HMP to optimize read retention and ITS-specific considerations as follows [44,45]. Raw sequencing data read quality was assessed with seqkit [46] and FastQC [47]. Primer sequences were removed with cutadapt [48]. The following processing was conducted with VSEARCH v2.23.0 [49]. Paired-end reads were merged and truncated at Phred score under 20, then merged reads were dereplicated and clustered into OTUs at 97% sequence similarity. De novo chimeras were removed with “uchime_denovo” and taxonomy was assigned with SINTAX using the Unite v9 eukaryotic database [50]. Feature table processing and analysis were conducted in R 4.3.1 using primarily the phyloseq [51], microViz [52], and vegan [53] packages as follows: nonfungal OTUs were removed, and fungal OTUs with unknown phyla were resolved by manual BLAST searches against the Unite v9 eukaryotic and NCBI nonredundant databases. When taxonomy could not be resolved at the phylum level or lower, the OTU was removed from further analysis. The final OTU table was rarefied to 1,500 read depth by taking the average of 1,000 rarefaction iterations using the EcolUtils package [54] and referred to as “rarefied feature table(s)” for downstream analysis.

### Sample size

Sample size was determined by the available data. The HMP cohort collected various biological samples from 300 donors [43]. Of these donors, fecal samples from 147 individuals were sequenced for ITS2 (mycobiome characterization) [14] and blood samples from 298 individuals were genotyped using whole-genome sequencing [15]. We matched individuals with paired ITS2 and whole-genome sequencing using the RANDSID variable from dbGaP accession phs000228.v4.p1 (*n* = 136). Sixteen individuals were removed during preprocessing of whole-genome sequencing (see below), leading to the final sample size of 125 individuals.

### Genome-wide association study

Genomic variants were accessed from dbGaP accession phs000228.v4.p1. Variant calling has been previously described [15]. To contextualize our findings in the light of previous bacterial microbiome GWAS, variant processing and statistical comparisons were aligned with the methods of previous studies where possible [7,15]. All processing was conducted in PLINK software [55,56]. Variants with over 5% missingness were removed, and self-reported sex was checked against imputed sex (no individuals were removed). Variants were filtered for minor allele frequency over 5% and for Hardy–Weinberg equilibrium chi-squared tests **P* *< 1^−10^. Sixteen individuals with high heterozygosity rates (over three times the standard deviation) were removed. To account for population stratification, independent variants (not in linkage disequilibrium) were extracted and used for principal component analysis. Aligning with previous bacterial GWAS methods to examine associations at each taxonomic level, the rarefied fungal feature table was collapsed at the phyla, order, class, family, genus, species, and OTU levels, taxa with less than 30% prevalence were removed, and the remainder were normalized with a center log ratio transformation. This resulted in the inclusion of 44 highly prevalent fungal taxa for the GWAS analysis. GWAS comparisons were made with the ‘—glm’ function for each combination of genomic variant and fungal taxa, and covariates included sex, extraction method used for WGS data (all ITS samples were extracted with similar methods, and all participants were sampled at one site), and the first 10 principal components of genetic stratification. Statistical significance of SNP–fungi associations was set at three thresholds; study-wise significance as FDR correction for all fungal taxa and all genomic variants at *Q* < 0.2, the standard GWAS genome-wide significance at *P* < 5 × 10^−8^, and an exploratory threshold that accounted for all genomic variants within one fungal taxa (*Q* < 0.05). QQplots were constructed for each fungal taxa to ensure *P* values were not artificially inflated, i.e., by population stratification. Genetic variants that met any significance threshold defined above were considered FAVs. FAVs were annotated with the SNPNexus web tool [57] using the GRCh37/hg19/b37 assembly.

### eQTL annotation

FAVs were probed for eQTL annotation and therefore association with differentially expressed genes across body tissues using the GTEx v10 database release (https://www.gtexportal.org/home/downloads/adult-gtex/qtl) on the GRCh38/hg38/b38 assembly. The GTEx lookup reference table was first used to confirm that the current hg19 dbSNP identifiers remained the same for the hg38 genomic build. FAV-eQTLs were mined from the list of significant variant-gene pairs in the *cis-*eQTL v10 GTEx release using the GTEx summary statistics for slope and *P* value.

### Phenome-wide associations

Phenome-wide associations to the FAVs were performed with the PheWEB online platform [30] and the TopMED-Imputed UKBiobank dataset. This dataset, publicly available, comprises 1,419 broad electronic health record codes from 51 to 78,000 cases and 167 to 407,000 controls. Phenotype associations with each FAV were considered significant at 3.52 × 10^−5^, a phenome-wide Bonferroni correction for the number of phenotypes tested at *α* = 0.05. Validation of two FAV associations with cardiovascular disease was performed with the ‘phewas’ function of the ieugwasr R package [31].

### Two-sample mendelian randomization

Two-sample mendelian randomization analysis (MR) was performed on each fungal taxa (exposure) and the associated FAVs (instruments) with the TwoSampleMR R package [58]. All FAVs were input as instruments and pruned to independent variants using the cutoff of *r*^2^ = 0.0001 and 1,000kb LD blocks (*n* = 11 independent instruments). Outcome GWAS were selected from the OpenGWAS database [31] that related to cardiovascular disease (*n* = 19) and subset to retain large (*n*_case_ > 20,000) GWAS that contained all 11 instruments (*n* = 8 outcome GWAS). Exposure and outcome SNPs were harmonized, then mendelian randomization was run for each exposure-outcome combination with default parameters and Bonferroni correction for multiple comparisons.

### *Kazachstania* in the HMP cohort

*Kazachstania* counts were aggregated at the genus level and center-log transformed. Then, the transformed abundance was compared to the genotypes for the two heart disease-associated FAVs using Kruskal–Wallis with Dunn’s post hoc test. Next, *Kazachstania* abundance and prevalence were summarized out of the HMP mycobiome dataset at the genus level. To compare mycobiome composition between individuals with and without *Kazachstania*, PERMANOVA was conducted on Bray–Curtis distances at the genus level, using sequencing depth as a covariate. Principal component analysis was conducted on center-log transformed counts at the genus level after removing samples with a sequencing depth under 1,000. The 8 fungal genera that contributed the most to the PCA loadings were plotted using the ord_plot() function in the microViz R package [52].

To compare *Kazachstania* across multiple human cohorts, ITS sequencing data were accessed from the Sequence Read Archive using a custom-built script (see Data availability statement). Raw sequencing data were downloaded with fastq-dump, then primers were removed if primer sequences were reported in each associated publication using cutadapt. OTUs were generated and taxonomy assigned using VSEARCH as described above for the HMP cohort and the Unite database. Only samples from healthy participants were used, i.e., the “control” arm of a cross-sectional study. Data from each study were iteratively rarefied according to the study’s average sequencing depth, then ordinated using center-log transformation at the genus level as described above. Mycobiome composition was compared between individuals with and without detectable *Kazachstania* using PERMANOVA on Bray–Curtis, genus-level distances if there were more than two individuals with *Kazachstania* in each dataset.

## Supporting information

S1 Fig Quantile-quantile plots of fungal taxa associations with independent SNPs. The dashed red line shows the expected distribution of *P* values under the null hypothesis, and black dots show the observed *P* values for each SNP. To reduce computational burden, independent SNPs are shown. Source code and data availability: https://doi.org/10.5281/zenodo.15659049.
(DOCX)

S2 Fig Linkage disequilibrium of FAVs. A density histogram of linkage disequilibrium values for genome-wide significant FAVs for each fungi, shown as *R*^2^ values (squared pairwise correlation, where *R*^2^ approaching 1 has high linkage disequilibrium) of all FAV-FAV pairwise combinations. The colors depict the chromosome of FAVs. Fungal taxa are shown that are associated with more than two FAVs. Source code and data availability: https://doi.org/10.5281/zenodo.15659049.
(DOCX)

S3 Fig Of the FAVs that overlap with genes, almost all are annotated as intronic and non-coding. A sunburst plot shows the relative proportions of intronic FAVs compared to FAVs of other potential functional consequence (3′ downstream, 5′ upstream, or 3′ untranslated region) for each FAV-overlapped gene (*CDH13*, *PTPRC*, *ANAPC10*, *NAV2*). Source code and data availability: https://doi.org/10.5281/zenodo.15659049.
(DOCX)

S4 Fig When *Kazachstania* is prevalent within a cohort, its presence is associated with an altered mycobiome composition. Raw mycobiome sequencing data was obtained from five published studies and re-analyzed under a uniform bioinformatics pipeline, then rarefied at varying levels depending on sequencing depth. Mycobiome composition is shown as genus-level Bray–Curtis distances and individual samples are colored based on whether *Kazachstania* was detected (blue) or not (gray). In the cohort PRJNA541487, mycobiomes with detectable *Kazachstania* significantly differ from those without (PERMANOVA *R*^2^ = 7.2, *P* = 0.005). Source code and data availability: https://doi.org/10.5281/zenodo.15659049.
(DOCX)

S1 Table Summary statistics for 148 FAVs. Source code and data availability: https://doi.org/10.5281/zenodo.15659049.
(XLSX)

S2 Table SNPNexus results for FAVs that overlap with genes. Source code and data availability: https://doi.org/10.5281/zenodo.15659049.
(XLSX)

S3 Table GTEx results for FAV-eQTLs. ‘tss_distance’ is the distance between the variant and transcription start site (positive = upstream); ‘af’ is allele frequency of alt allele; and ‘pval_nominal’ and ‘slope’ are GTEx QTL testing results. Source code and data availability: https://doi.org/10.5281/zenodo.15659049.
(XLSX)

S4 Table Metadata corresponding to mycobiome cohorts (S4 Fig). Source code and data availability: https://doi.org/10.5281/zenodo.15659049.
(XLSX)

S5 Table Linear model of *Kazachstania* relative abundance and clinical/demographic variables. Source code and data availability: https://doi.org/10.5281/zenodo.15659049.
(XLSX)

## Abbreviations:

FAV: fungi-associated variant
FDR: false discovery rate
GTEx: Genotype-Tissue Expression
GWAS: genome-wide association study
HMP: Human Microbiome Project
OTU: operational taxonomic unit
