# Supplementary material for: Gut fungi are associated with human genetic variation and disease risk
Source: PLoS Biol. 2025 Sep 2;23(9):e3003339. doi: 10.1371/journal.pbio.3003339 (PMC12404459; doi:10.1371/journal.pbio.3003339)
Supplement: S1 Fig — The dashed red line shows the expected distribution of P values under the null hypothesis, and black dots show the observed P values for each SNP. To reduce computational burden, independent SNPs are shown. Source code and data availability: https://doi.org/10.5281/zenodo.15659049. (DOCX) [file pbio.3003339.s001.docx]

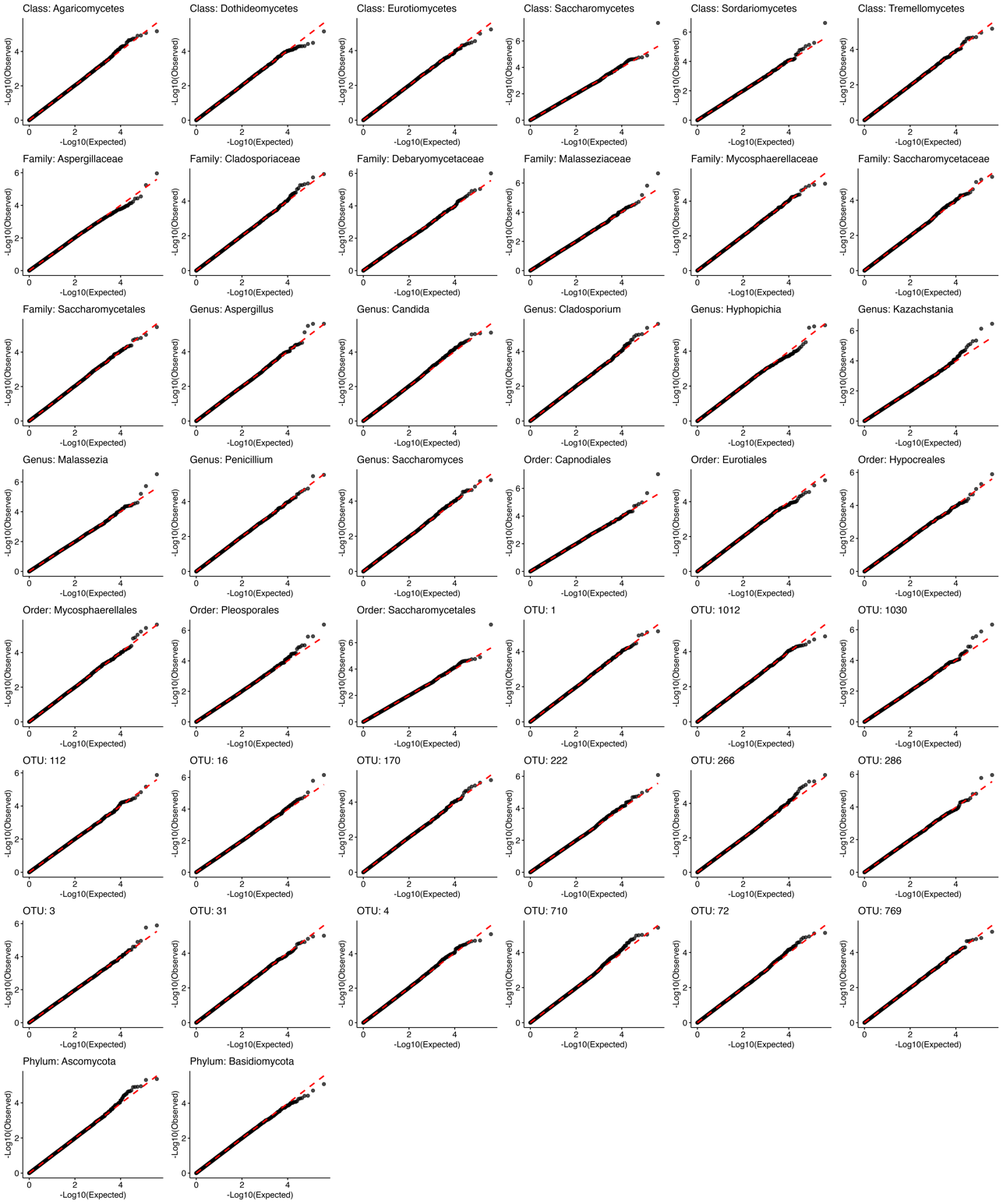
**S1 Fig: Quantile-quantile plots of fungal taxa associations with independent SNPs.** The dashed red line shows the expected distribution of P values under the null hypothesis, and black dots show the observed *P* values for each SNP. To reduce computational burden, independent SNPs are shown. Source code and data availability: https://zenodo.org/records/15659050
