# Supplementary material for: Gut fungi are associated with human genetic variation and disease risk
Source: PLoS Biol. 2025 Sep 2;23(9):e3003339. doi: 10.1371/journal.pbio.3003339 (PMC12404459; doi:10.1371/journal.pbio.3003339)
Supplement: S2 Fig — A density histogram of linkage disequilibrium values for genome-wide significant FAVs for each fungi, shown as R2 values (squared pairwise correlation, where R2 approaching 1 has high linkage disequilibrium) of all FAV-FAV pairwise combinations. The colors depict the chromosome of FAVs. Fungal taxa are shown that are associated with more than two FAVs. Source code and data availability: https://doi.org/10.5281/zenodo.15659049. (DOCX) [file pbio.3003339.s002.docx]

**S2 Fig: Linkage disequilibrium of FAVs.** A density histogram of linkage disequilibrium values for genome-wide significant FAVs for each fungi, shown as R^2^ values (squared pairwise correlation, where R^2^ approaching 1 has high linkage disequilibrium) of all FAV-FAV pairwise combinations. The colors depict the chromosome of FAVs. Fungal taxa are shown that are associated with more than two FAVs. Source code and data availability: https://zenodo.org/records/15659050

**
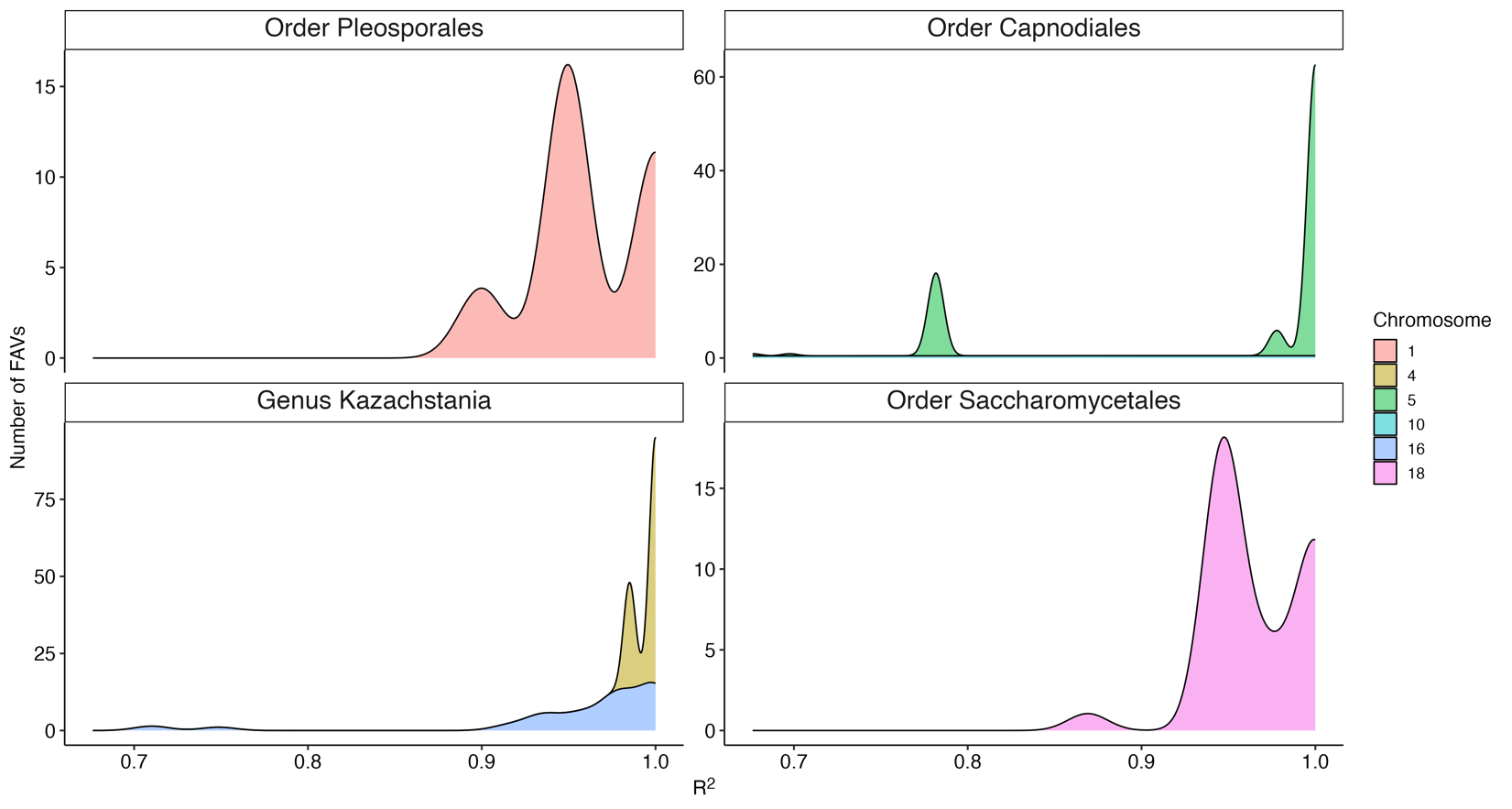
**
