# Supplementary material for: Gut fungi are associated with human genetic variation and disease risk
Source: PLoS Biol. 2025 Sep 2;23(9):e3003339. doi: 10.1371/journal.pbio.3003339 (PMC12404459; doi:10.1371/journal.pbio.3003339)

**S3 Fig: Of the FAVs that overlap with genes, almost all are annotated as intronic and non-coding.** A sunburst plot shows the relative proportions of intronic FAVs compared to FAVs of other potential functional consequence (3’ downstream, 5’ upstream, or 3’ untranslated region) for each FAV-overlapped gene (*CDH13*, *PTPRC*, *ANAPC10*, *NAV2*). Source code and data availability: https://zenodo.org/records/15659050


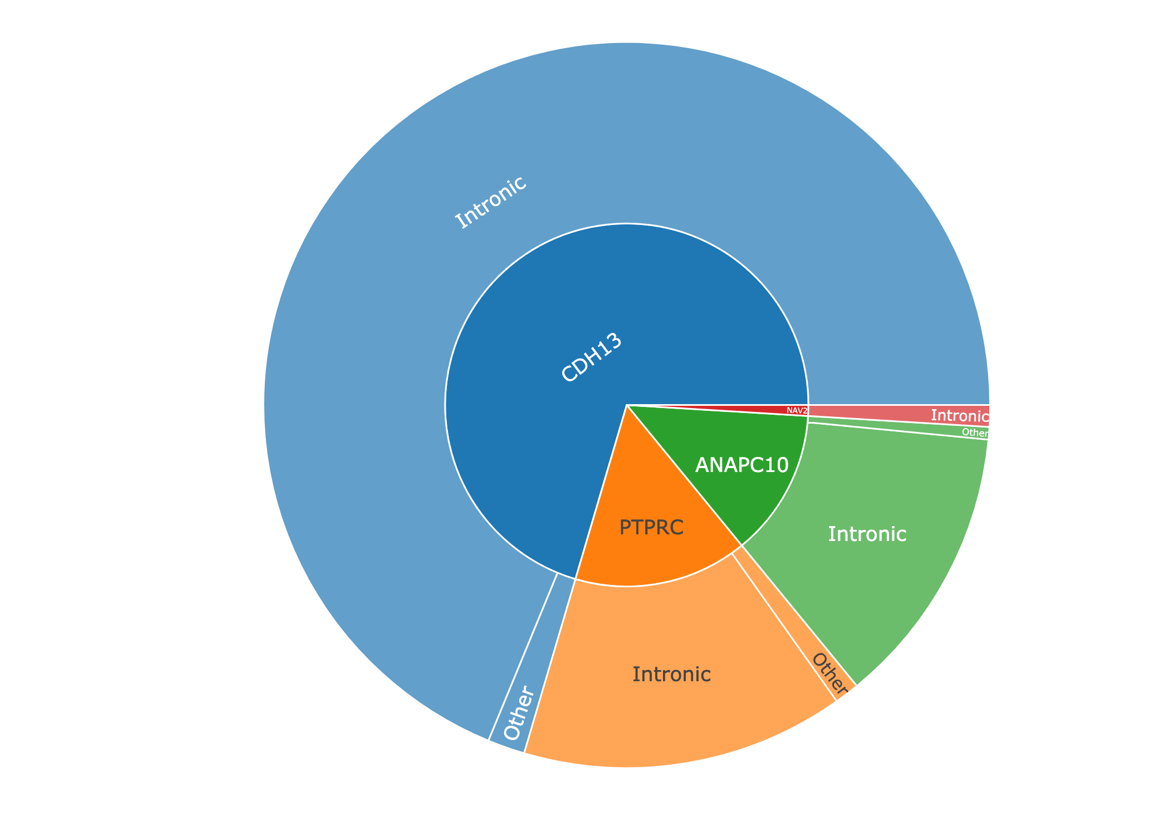

Supplement: S3 Fig — A sunburst plot shows the relative proportions of intronic FAVs compared to FAVs of other potential functional consequence (3′ downstream, 5′ upstream, or 3′ untranslated region) for each FAV-overlapped gene (CDH13, PTPRC, ANAPC10, NAV2). Source code and data availability: https://doi.org/10.5281/zenodo.15659049. (DOCX) [file pbio.3003339.s003.docx]
