# Supplementary material for: Gut fungi are associated with human genetic variation and disease risk
Source: PLoS Biol. 2025 Sep 2;23(9):e3003339. doi: 10.1371/journal.pbio.3003339 (PMC12404459; doi:10.1371/journal.pbio.3003339)

**S4 Fig: When *Kazachstania* is prevalent within a cohort, its presence is associated with an altered mycobiome composition.** Raw mycobiome sequencing data was obtained from five published studies and re-analyzed under a uniform bioinformatics pipeline, then rarefied at varying levels depending on sequencing depth. Mycobiome composition is shown as genus-level Bray-Curtis distances and individual samples are colored based on whether *Kazachstania* was detected (blue) or not (grey). In the cohort PRJNA541487, mycobiomes with detectable *Kazachstania* significantly differ from those without (PERMANOVA R^2^=7.2, *P* = 0.005). Source code and data availability: https://zenodo.org/records/15659050


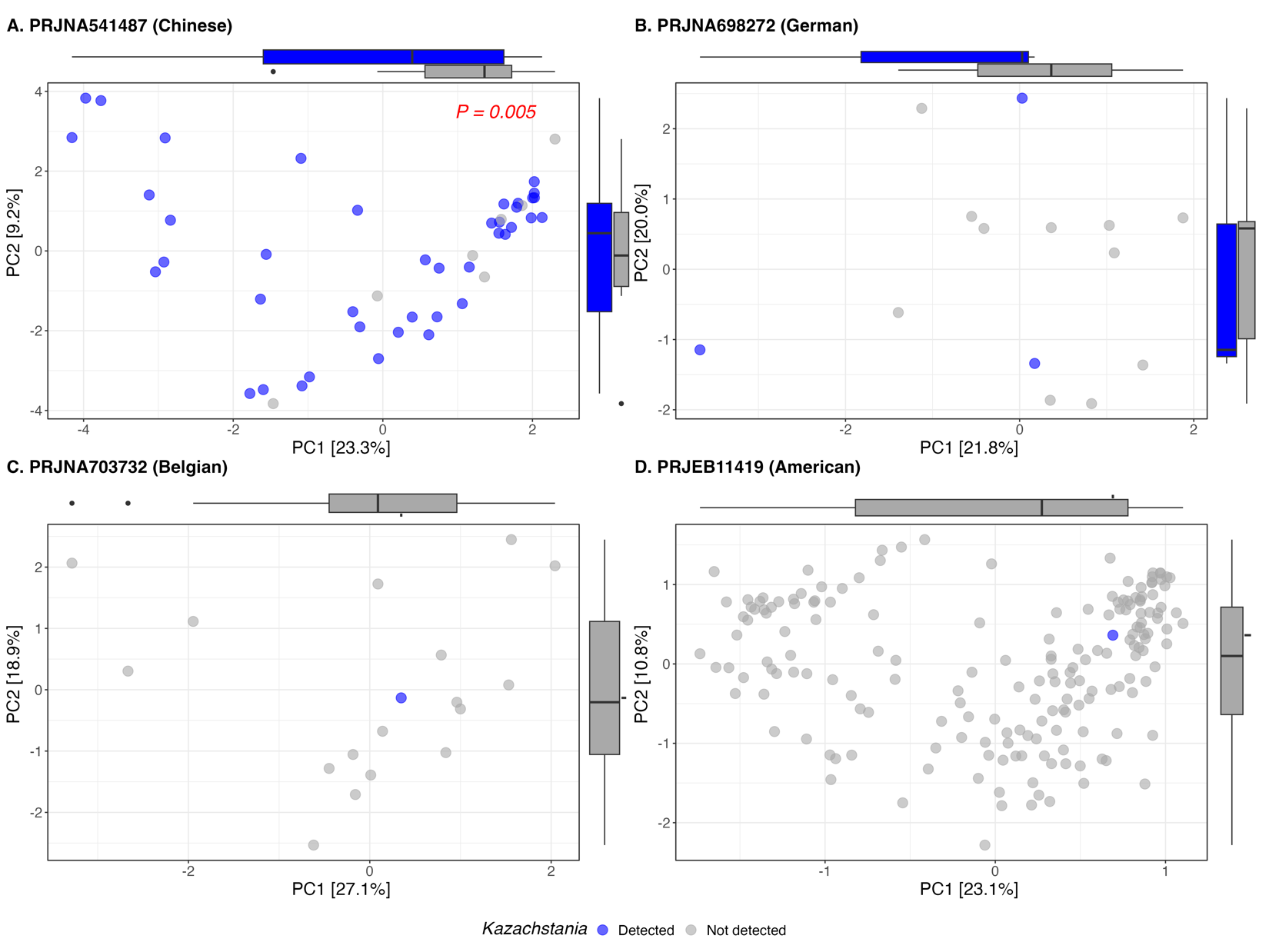

Supplement: S4 Fig — Raw mycobiome sequencing data was obtained from five published studies and re-analyzed under a uniform bioinformatics pipeline, then rarefied at varying levels depending on sequencing depth. Mycobiome composition is shown as genus-level Bray–Curtis distances and individual samples are colored based on whether Kazachstania was detected (blue) or not (gray). In the cohort PRJNA541487, mycobiomes with detectable Kazachstania significantly differ from those without (PERMANOVA R2 = 7.2, P = 0.005). Source code and data availability: https://doi.org/10.5281/zenodo.15659049. (DOCX) [file pbio.3003339.s004.docx]
